# Daily temperature fluctuations unpredictably influence developmental rate and morphology at a critical early larval stage in a frog

**DOI:** 10.1186/1472-6785-13-18

**Published:** 2013-05-04

**Authors:** Juliana M Arrighi, Ezra S Lencer, Advait Jukar, Daesik Park, Patrick C Phillips, Robert H Kaplan

**Affiliations:** 1 Department of Biology, Reed College, 3203 SE Woodstock Blvd, Portland, OR 97202, USA; 2 Systems Science, Portland State University, Portland, OR, USA; 3 Ecology and Evolutionary Biology, Cornell University, Ithaca, NY, USA; 4 Environmental Science and Policy, George Mason University, Fairfax, VA, USA; 5 Division of Science Education, Kangwon National University, Chuncheon, Kangwon, 200-701, South Korea; 6 Institute of Ecology and Evolution, University of Oregon, Eugene, OR, 97403, USA

**Keywords:** *Bombina orientalis*, Amphibian, Diel temperature fluctuation (DTF), Developmental plasticity, Thermal performance curve, Reaction norm

## Abstract

**Background:**

Environmental temperature has profound consequences for early amphibian development and many field and laboratory studies have examined this. Most laboratory studies that have characterized the influence of temperature on development in amphibians have failed to incorporate the realities of diel temperature fluctuations (DTF), which can be considerable for pond-breeding amphibians.

**Results:**

We evaluated the effects of different ecologically relevant ranges of DTF compared with effects of constant temperatures on development of embryos and larvae of the Korean fire-bellied toad (*Bombina orientalis*). We constructed thermal reaction norms for developmental stage, snout- vent length, and tail length by fitting a Gompertz-Gaussian function to measurements taken from embryos after 66 hours of development in 12 different constant temperature environments between 14°C and 36°C. We used these reaction norms as null models to test the hypothesis that developmental effects of DTF are more than the sum of average constant temperature effects over the distribution of temperatures experienced. We predicted from these models that growth and differentiation would be positively correlated with average temperature at low levels of DTF but not at higher levels of DTF. We tested our prediction in the laboratory by rearing *B. orientalis* embryos at three average temperatures (20°C, 24°C, and 28°C) and four levels of thermal variation (0°C, 6°C, 13°C, and 20°C). Several of the observed responses to DTF were significantly different from both predictions of the model and from responses in constant temperature treatments at the same average temperatures. At an average temperature of 24°C, only the highest level of DTF affected differentiation and growth rates, but at both cooler and warmer average temperatures, moderate DTF was enough to slow developmental and tail growth rates.

**Conclusions:**

These results demonstrate that both the magnitude of DTF range and thermal averages need to be considered simultaneously when parsing the effects of changing thermal environments on complex developmental responses, particularly when they have potential functional and adaptive significance.

## Background

The relationship between organismal form and function depends on environmental context. For example, developmental rate in ectotherms is contingent on external temperature, and changes in developmental rate can in turn alter the relative allometries of different morphological and physiological traits [1]. The vast majority of laboratory studies that infer the effects of environment on development and physiology have been undertaken in constant environments. Yet the thermal environments encountered in nature can be quite variable, and it is unclear whether patterns predicted with models based on observations from constant environments provide an adequate representation of this variation.

A small but growing number of studies have addressed the effects of diel temperature fluctuation (DTF) on amphibian development, which is known to be particularly sensitive to variable environmental inputs [2-6]. The implications of this body of work are multifaceted and include the observation that small increases in average global temperatures may have measurable effects on performance in amphibian embryos and larvae. However, the specific effects of associated daily and seasonal extremes on early amphibian development are less well understood. We contribute to the exploration of the differing developmental responses to environmental variability, focusing on realistic DTF during embryonic development, a period when phenotypic plasticity in response to temperature variation is considered ubiquitous [7-9].

For an embryo developing in a constant temperature environment, a measure of a phenotypic characteristic (e.g., developmental stage or body size) at a given time point can be represented by the functional response of the animal to that temperature, *z*(*T*) [10]. When temperature varies, the phenotype will be a complex function of the particular temperature path each individual experiences taken over the age- and/or stage-specific effects of each temperature,$\zeta \left(\bar{T}\right)$. In other words, temperatures experienced at one time point might affect the individual’s response to different temperatures experienced at later time points (i.e., there will be carryover effects). For instance, an individual experiencing a transient temperature exceeding some critical thermal stress limit might experience prolonged period of slowed growth relative to an individual exposed to a lower constant temperature, even if both individuals eventually experience the same average temperature. If variability *per se* has no effects beyond those caused by the instantaneous effects of temperature experienced at each moment (i.e., there are no carryover effects), then the expected phenotype should simply be a function of the distribution of temperatures experienced, *f*(*T*) [10] and the functional response observed at each fixed temperature:

(1) $$\zeta \left(\bar{T}\right)=\int f\left(T\right)z\left(T\right)\mathrm{dT};$$

where the integral is taken over the range of observed temperatures (see also [6,11]). Equation (1) therefore provides a null model against which the hypothesis that temperature variation is more than the sum of individual temperature effects can be tested.

Much of the work on the effects of temperature fluctuations on development has been concerned with predicting developmental rates for ectotherms. Both models and empirical data have shown that temperature fluctuations can increase, decrease, or have no effect on developmental rates [6,12-20]. An emerging conclusion from such studies has been that temperature fluctuations are ecologically important when individuals experience temporary exposure to temperatures at the limits of their physiologically relevant thermal range [13,16,18,21] and that fluctuations at cooler versus warmer temperatures can have different or opposing effects on developmental traits [12,16].

Amphibian development shows a remarkable amount of plasticity throughout both the embryonic and larval periods (e.g., [22,23]). The timing of hatching and larval morphology at hatching can be influenced by a variety of maternal and external-environmental effects [24-26]. Among these environmental effects, temperature is perhaps the most pervasive ([27]; but see [28] and [29] for a discussion of the importance of maternal effects *per se*). Temperature effects drive adaptation on small geographic scales and short periods of time [30,31] and influence the timing and effectiveness of other environmentally cued hatching mechanisms [32].

At a proximate level, temperature affects the cellular and biochemical rates of differentiation and growth during development [33-35]. At the whole-organism level, environmental temperatures can interact with an individual’s genome to affect the amount of time spent developing, body shape, body size, and mass at metamorphosis, as well as trait values for a variety of other phenotypic characters at both the larval and adult stages (e.g. [23,36]). For example, in the frog *Limnodynastes peronii* (Duméril and Bibron, 1841) and the newt *Triturus alpestris* (Laurenti, 1768), DTF increased developmental rate and was shown to positively affect individual performance relative to developing in a constant temperature environment [3,4]. In *L. peronii*, DTF also increased tadpole body length measurements and decreased metamorph mass, indicating that fluctuating temperature environments can also affect morphology and body size.

Similarly, in the frog *Bombina orientalis* (Boulenger, 1890), warmer developmental temperatures have been shown to cause tadpoles to hatch at a younger developmental stage [2,37]. Moreover, warmer temperatures result in hatchling tadpoles with shorter tail lengths but longer snout-vent lengths [2,38]. Further, embryos experiencing greater amounts of temperature fluctuation in natural breeding ponds tended to hatch at a younger developmental stage and had longer snout-vent length measurements and shorter tail length measurements. These traits have been shown to affect tadpole locomotor performance and fitness at hatching stage, since hatchlings with greater tail length to snout-vent length ratios swim faster and have a higher probability of surviving predation [2,39]. Thus, for *B. orientalis*, a heterogeneous thermal environment can induce variation in traits associated with fitness within a population [2].

We combine two approaches, an empirical modelling approach and a comparative experimental approach. Specifically, we (1) establish thermal reaction norms to constant temperature variation in order to generate null models of developmental response in early larval development in *B. orientalis*, and then (2) use comparative, controlled diel temperature variation of different magnitudes to investigate whether observed effects are the result of simple additive DTF or whether particular thermal events have unexpected consequences.

## Methods

*Bombina orientalis* is widely distributed across the Korean Peninsula and ranges into parts of north-eastern China. Breeding populations of *B. orientalis* utilize a variety of permanent and semi-permanent water bodies as oviposition sites, including rice paddies and granite pools along mountainside streams. These breeding sites can differ remarkably in size, depth, permanence, and shading [37]. As a result, the thermal environment experienced among egg masses within and among populations can be highly variable [36] with respect to both average temperature and diel temperature fluctuation (DTF; [2])

### Breeding and egg collections

During the summer of 2006, adult *B. orientalis* were collected from a rice paddy outside the village of Duc Doo Won near the city of Chuncheon in the Province of KangWon-do in South Korea and maintained as part of a laboratory colony at Reed College in Portland, Oregon. All adults were housed at a constant 24°C and fed crickets *ad libitum* for at least 10 weeks after a prior breeding [37]. Male and female *B. orientalis* pairs were injected intraperitonealy with 250 IU and 375 IU, respectively, of human chorionic gonadotropin [37] and allowed to oviposit overnight. Fertilized eggs were collected the following morning at blastula stage (Gosner [40] stage 8) and placed in glass fingerbowls filled with 20% Holtfreter’s solution in one of the thermal treatments. Fingerbowls were loosely covered to prevent evaporative cooling and illuminated with an LED light that was set to a 12 h light 12 h dark schedule. Multiple sibships were pooled and randomly distributed across treatments.

### Design of thermal environments

We raised *B. orientalis* embryos in a total of 16 different constant and fluctuating thermal environments constructed in programmable incubators (HotPack model 317512 with a Watlow F4 controller). Temperature treatments ranged across those naturally experienced by *B. orientalis* in the field. Thermal maxima and minima were kept within the limits of 10°C and 36°C, since temperatures outside of that range have been observed to cause increased mortality (see [2]). Within each treatment, two data loggers (Onset Hobo data loggers) per treatment measured the water temperature in two different fingerbowls every 15 minutes. Temperature profiles between bowls were not statistically different.

### Development under constant temperatures

In order to assess the thermal reaction norm of an individual over an environmental gradient, organisms are generally maintained at a range of constant environmental conditions in the laboratory [6,10]. Over three occasions, twelve constant temperature environments were employed at temperatures between 14°C and 36°C at 2°C increments (i.e. 14, 16, … 34, 36°C). Forty embryos were distributed across fingerbowls in each treatment, with 10 embryos per bowl in four large fingerbowls so that 480 embryos were used in this phase of the study. The data from this portion was then used to generate models predicting growth and development under fluctuating temperatures as described in the modelling methods section.

### Development under fluctuating temperatures

**Figure 1:**
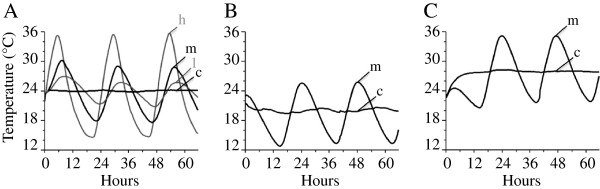
**Profiles of fluctuating temperature environments experienced by embryos in this study. **Temperature was monitored every 15 minutes and averaged from two data loggers in each thermal environment. Panel **A **shows a matched set of four treatments with average temperatures of 24°C. Panels **B **and **C **each show a matched set of two treatments with average temperatures of 20°C and 28°C, respectively. Lowercase letters refer to the magnitude of DTF in each treatment: constant temperature (**c**), low variation (**l**), medium variation (**m**), or high variation (**h**). See text for a more detailed description of these treatments.

In order to explore the effects of daily thermal fluctuation, embryos were allowed to develop under eight environmental treatments at four levels of DTF organized into three groupings by average temperature as shown in Figure 1 (panels A, B, C). This includes constant temperature treatments at each average temperature, which were repeated so that the statistical comparisons (described in the statistical methods section) were made against matched sets of randomized embryos. Twenty-four bowls were placed in each thermal condition, with up to 7 tadpoles in each bowl. In the case of the constant temperature treatment at 24°C, a controlled temperature room was utilized (24°C approx. +/- 0.5°C; Figure 1A). The fluctuating temperature treatments at 24°C included a low DTF environment (24°C approx. +/- 3°C), a medium DTF environment (24°C approx. +/- 6°C) and a high DTF (24°C approx. +/- 10°C). These three thermally varying environments were established within a single incubator that was programmed to ramp between 14°C and 38°C every 24 hours with a variable amount of Styrofoam insulation. Fingerbowls in the low temperature variation treatment group (l) were insulated with a Styrofoam cooler, fingerbowls in the medium temperature variation treatment group (m) were insulated with a plastic box and Styrofoam packing material, and fingerbowls in the high temperature variation treatment group (h) were placed in the incubator without any insulation. At average temperatures of each 20°C and 28°C DTF treatments were created using the same incubator profile, decreased or increased by 4°C, in separate incubators run synchronously. The fingerbowls in these treatments were insulated in the same way as medium variation treatment from the 24°C grouping. DTF treatment profiles for the second two groupings are shown in the second two panels (average 20°C and 28°C, respectively; Figure 1B, C).

### Measurement of developmental stage and morphology

**Figure 2:**
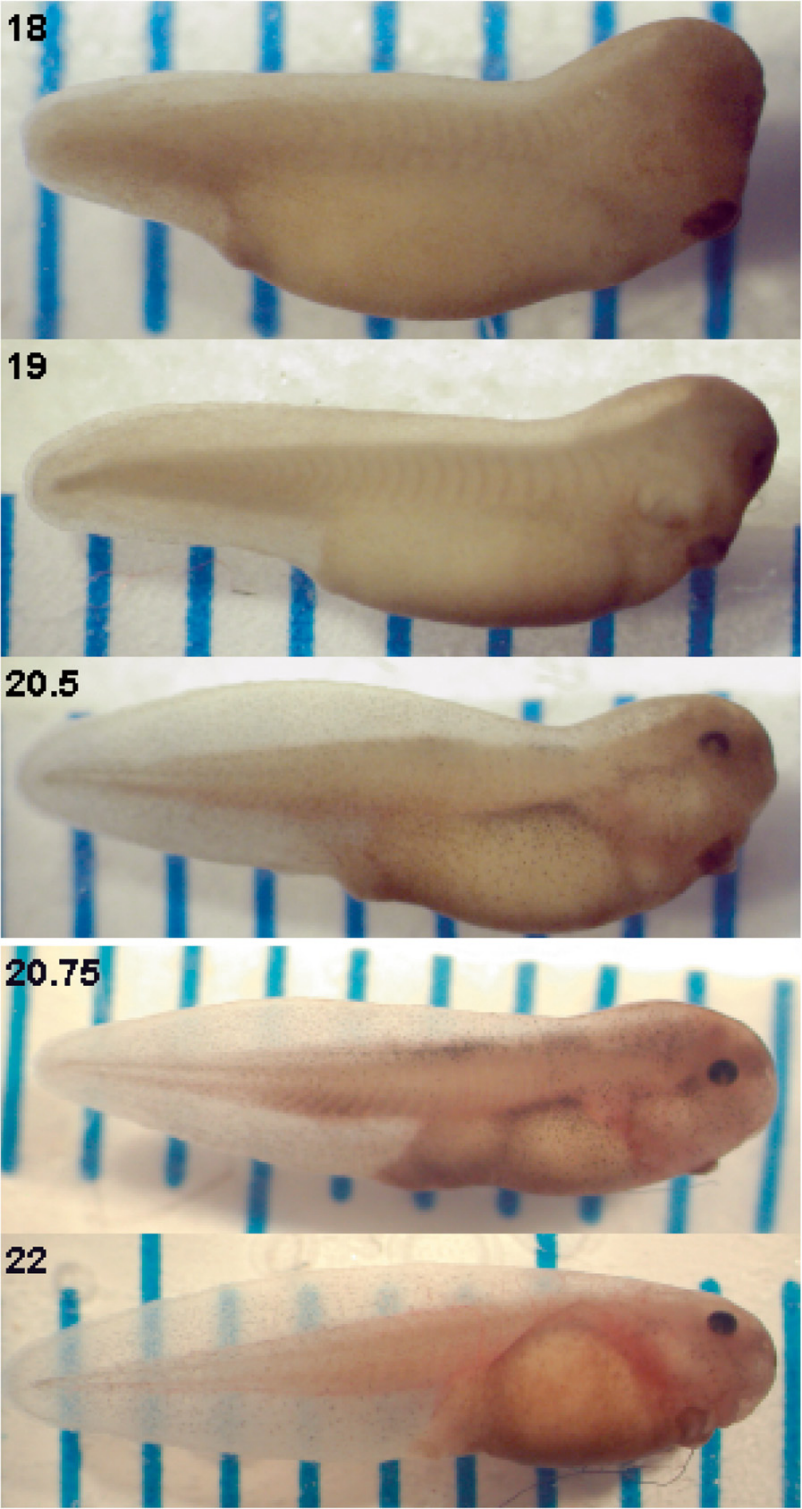
**Representative individuals at relevant developmental stages. **Tadpoles were photographed alongside a mm ruler and labelled with Gosner [40] stage. We also divided stages 20 to 21 into substages based on level of eye transparency [2]. Stages were defined by the following criteria. 19: no blood circulation in the gills; 20: blood circulation in the gills and minimal corneal transparency (not shown); 20.25: corneal transparency extending 1/4 way down eye (not shown); 20.5: corneal transparency extending 1/2 way down eye; 20.75:corneal transparency extending 3/4 way down eye; 21: cornea fully transparent (not shown), 22: tailfin transparent and circulation observed.

Studies of temperature and development in amphibians often use life history stages as observational markers. We use the developmental stage range where hatching occurs in the field. There are many environmental signals that influence the time and stage at which tadpoles hatch (e.g., [32,41]). In field populations, *Bombina orientalis* can hatch as early as Gosner [40] stage 19, but most hatching occurs during the time when the cornea becomes transparent between Gosner stages 20 and 21 ([39,40]; Figure 2). In laboratory environments *B. orientalis* embryos reach field hatching stages after 66 hours of development at constant 24°C but often do not hatch until later stages (personal observation). Because of this observation, embryos in all experiments described here were allowed to develop in all temperature treatments for 66 hours, at which point they were removed from temperature treatments, manually hatched if necessary, photographed and staged under a dissection microscope.

In all cases, for both constant and DTF experiments, at 66 hours of development tadpoles were staged and photographed alongside a mm ruler using an Olympus digital camera interfaced to a stereomicroscope. Embryos and larvae were staged after Gosner [40], with the following modification. Stages 20 to 21 were further subdivided into 4 stages corresponding to the degree of corneal transparency ([39,42]; Figure 2).

Morphological measurements were then taken from photographs in either Adobe Photoshop or ImageJ [43] after checking for scaling artifacts between the software. We measured total length, defined as the length from the tip of the snout to the end of the tail, snout- vent length, defined as the length from the tip of the snout to a point at the center of the tadpole perpendicular to the posterior margin of the vent, and tail length defined as the length from center of the tadpole perpendicular to the posterior margin of the vent to the tip of the tail, all with respect to the ruler photographed with the tadpole.

### Modelling methods

Mean responses from development under constant temperature experiments were used to estimate the temperature response function for each snout-vent length, tail length, and stage (*z*(*T*); Equation 1). Because thermal reaction norms typically fall off beyond critical thermal maxima and minima, we fit each reaction norm using several different nonlinear functions, distinguishing among the models using the Akaike Information Criterion [6,44]. We compared three different functions: quadratic, Gaussian, and Gompertz-Gaussian, respectively specified by

$$\begin{array}{l} \mathrm{zT}=aT^{2}+\mathrm{bT}+c\\ \mathrm{zT}=a\exp\left(-{\left(T-b\right)}^{2}/c\right)\\ \mathrm{zT}=a\exp\left(-e^{b}{{}^{(}}^{T}{{}^{-}}^{c}{}^{)-8}-{\left(T-c\right)}^{2}/d\right) \end{array}$$

Other nonlinear functions suggested by [6] (modified Guassian, Weibull and beta) did not yield convergent solutions for our data. In contrast, the Gompertz-Gaussian suggested by Martin and Huey [11] performed much better and provided an extremely good fit to the data. The predicted effects of thermal variation over these thermal reaction norms (Equation 1) was calculated using the convolution of the estimated thermal reaction norm and the distribution of the thermal variation profiles for each temperature treatment assumed to be uniform for simplification.

All calculations were performed using Mathematica, with the nonlinear fitting and associated statistics being calculated using the NonLinearModelFit function [45]. Errors on the predicted responses were calculated using a bootstrapping approach in which individual temperature-dependent responses were resampled with replacement and then used to recalculate the best-fitting reaction norm. The data were resampled 10,000 times and used to construct an error distribution from which the standard error was calculated.

### Statistical methods

In all cases, fingerbowl averages rather than raw measurements of individual were used to avoid any issues related to pseudo-replication, and we conducted all analyses using the mean value for each fingerbowl. All statistical tests were performed with JMP Version 7 [46]. Data were checked for normality and no transformations were found to be necessary. To test for the effect of DTF we investigated each average temperature separately. We tested whether our DTF treatments were significantly different from their matched constant temperature treatment. Temperature variation was entered as a two or four level fixed effect. In all instances we conducted an ANOVA to test for significant differences among our treatments and a posteriori tests using Tukey-Kramer LSD.

## Results

### Modelling effects of DTF based on observations at constant temperatures

**Figure 3:**
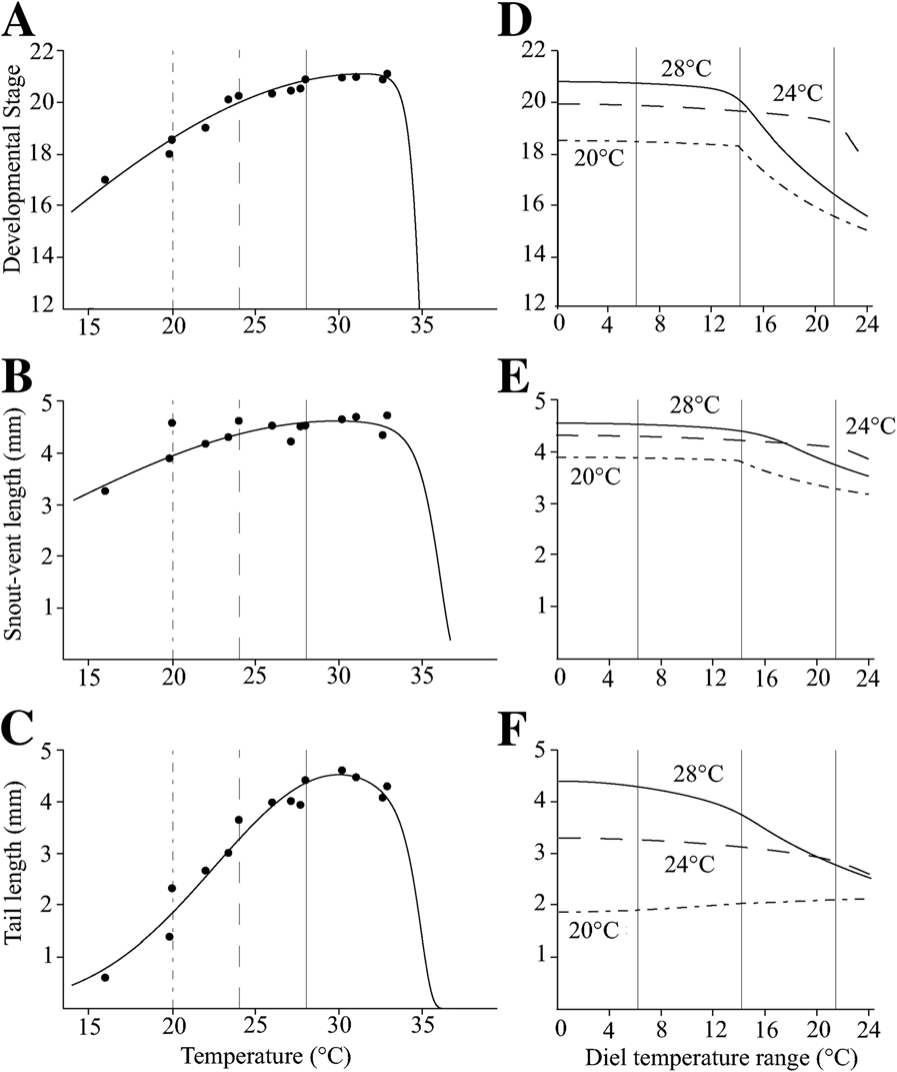
**Constant temperature reaction norms and predictions across fluctuating temperature ranges for three average temperatures. **Points in panels **A**-**C** show means for in measured response variables in each constant temperature after 66 hours. In each case, the best fitting model for the reaction norm was a Gompertz-Gaussian function (*R*^2^ > 0.99)*, *using parameters in Table 1 and described in the text. Vertical lines on panels **A**-**C** are drawn at average temperatures used in variable temperature treatments (solid: 28°C average; dashed: 24°C average; dot-dash: 20°C average). Panels **D**-**F **show the predictions for the variable temperature experiments, assume a uniform distribution of temperatures within the diel variation range and integrating the reaction norms over the experimentally induced environmental variation (Equation 1). Vertical lines in panels **D**-**F** indicate approximate temperature range locations of the 6°C, 14°C, and 22°C variation treatments in the DTF experiments (**l**, **m**, and **h** in Figure 1). Error bars show +/- 1 standard error.

Embryos raised over the range of constant temperatures from 14 to 36°C displayed thermal reaction norms for developmental stage, snout-vent length and tail length that are largely typical for ectotherms (Figure 3). Developmental rate was much lower at temperatures below 16°C, and at 14°C embryos only developed to early neurulation (Figure 3A). Since snout-vent length and tail length are defined in terms of an embryo’s vent, those measurements cannot be measured until the structure develops, which occurs at early tail bud stage (stage 17) at 66 hours into development at 16°C. Constant temperatures of 36°C were lethal for embryos, which was interpreted as having a value of zero for all measures when fitting the data. Developmental rate after 66 hours, as a function of temperature, increases monotonically until approximately 34°C when it plateaus and then abruptly declines. Snout-vent length behaves similarly (Figure 3B), but tail length shows a decline in growth rate beginning at temperatures of 30°C (Figure 3C). The reaction norms are well fit by the Gompertz-Gaussian model, but not by Gaussian or quadratic models (coefficients of determination of the fit models exceed 0.99 for all traits; Table 1).

**Table 1:** Model statistics and parameters

	**Stage**	**Snout-vent length**	**Tail length**
*Quadratic*			
AIC	1080.7	446.6	202.3
*Gaussian*			
AIC	1039.6	403.4	208.9
*Gompertz-Gaussian*			
AIC	298.5	224.7	93.8
_*R*_2	0.99	0.99	0.99
*A*	21.13	4.55	4.62
*B*	2.33	1.60	1.31
*C*	31.72	30.10	30.33
*D*	1068.82	112.95	654.34

Model selection and parameter fitting for the functional reaction norms. In each case, the Gompertz-Gaussian provided the best fit (as indicated by the low AIC value).

The fit reaction norms (Figure 3, A-C) can be used to predict developmental outcomes for each trait over continuously varying levels of DTF for the three different average temperatures and DTF’s used in our experiments (Figure 3, D-F). Using this approach, developmental rate is predicted to be relatively insensitive to DTF at the average temperature of 24°C until a DTF range of approximately 20°C is exceeded (Figure 3D). However, DTF range greater than 13°C is expected to influence developmental rate to 66 hours at both cooler (20°C) and warmer (28°C) average temperatures. A similar pattern is observed for snout-vent length (Figure 3E) where non-linear effects of DTF are not predicted at an average of 24°C until DTF spans of approximately 20°C (Figure 3E), whereas at average temperatures of 20°C and 28°C effects emerge at smaller DTF ranges. In contrast, based on the responses to constant temperature environments, increased DTF is predicted to barely change tail growth relative to the average temperature response for constant low temperatures, but to lead to decreased growth as the average constant temperature increases (e.g., 28°C; Figure 3F).

### Description of DTF treatments

For treatments with average temperatures of 24°C the mean temperatures for all four environments were within 0.24°C of each other (Figure 1A). The high DTF treatment extended between 9°C and 11°C above and below the mean. It did not reach 36°C, where 100% mortality was observed in the constant temperature treatment. At both the averages of 20°C (Figure 1B) and 28°C (Figure 1C), DTF treatments approximated the medium fluctuation level of the average 24°C experiment, extending between 6°C and 7°C above and below the mean. The shapes of the three medium fluctuation profiles were similar (“m” in Figure 1, A-C). The 20°C and 28°C constant temperature treatments and their matched, medium DTF treatment groups also had similar average temperatures differing by 1.18°C and 0.83°C, respectively.

### Comparative experimental effects of DTF on developmental stage

**Figure 4:**
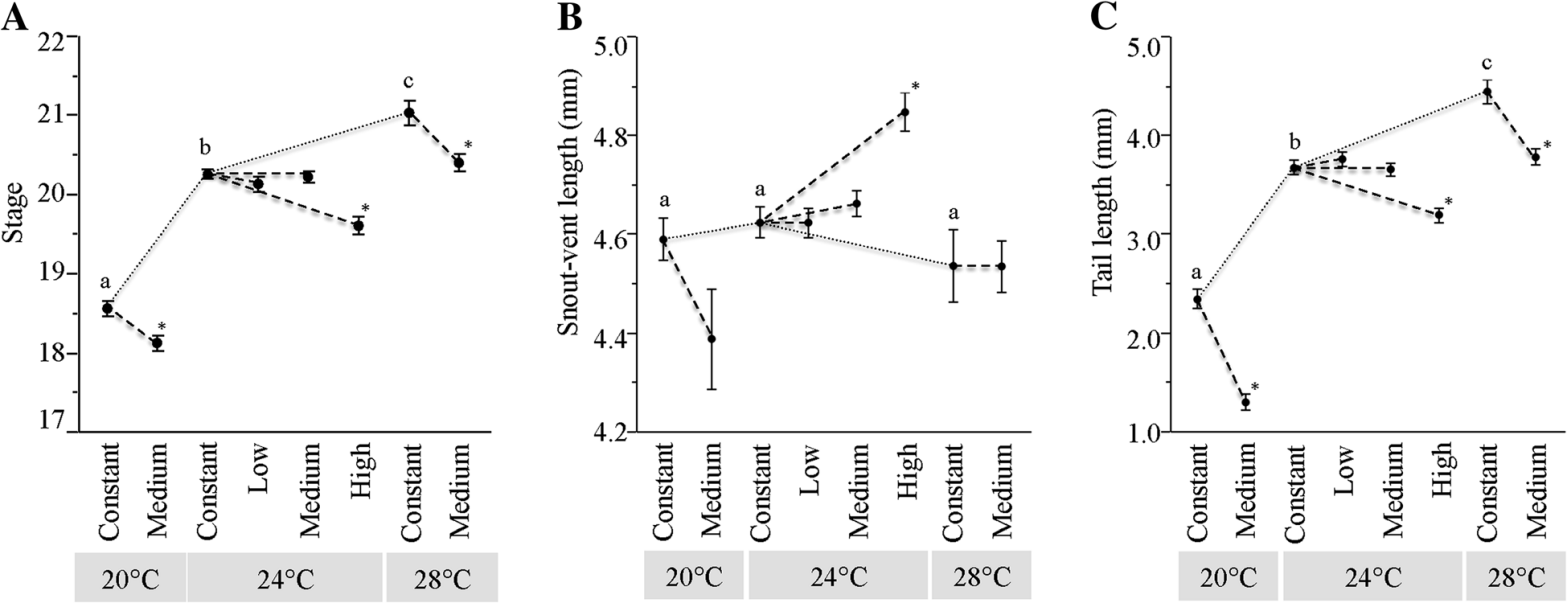
**Effects of average temperature and DTF. **Effects shown are for developmental stage (**A**), snout-vent length (**B**), and tail length (**C**). Observations were from 66 hours of development in DTF environments with dotted lines connect constant treatments, and dashed lines connect response to fluctuating temperature with response to matched constant temperature. Lower case letters denote significant difference among constant temperatures (Tukey HSD *P* = < 0.05). Stars denote variable treatments with mean trait values that are significantly different (Tukey HSD *P* = < 0.05) from the matched constant temperature environment. Error bars show +/- 1 standard error.

When development was terminated after 66 hours, all embryos or hatched larvae were between Gosner [40] stages 18 and 22 (within the range of stages that *B. orientalis* are known to hatch at in the field; [39]; and Figure 2). When tadpoles from constant temperature environments were compared to each other, the embryos from warmer constant temperature environments reached a later developmental stage in the same period of time (i.e., 66 hours; Figure 4A; *F*_2, 62_ = 81.36, *P* < 0.01).

We tested for the effect of variable DTF on developmental stage by investigating each average temperature treatment separately. At 24°C increasing DTF had a significant effect on stage (*F*_3, 87_ = 11.68, *P* < 0.01), but only tadpoles that developed in the high variation treatment were found to be at an earlier developmental stage at 66 hours (Figure 4A; Tukey HSD, *P* < 0.05). In contrast, at both 20°C and 28°C, embryos raised in medium DTF were less differentiated after 66 hours of development than tadpoles from their respective constant temperature treatments (Figure 4A; 20°C, *F*_1, 42_ = 11.23, *P* < 0.01; 28°C: *F*_1, 42_ = 4.46, *P* < 0.05).

### Comparative experimental effects of DTF on morphology

In contrast to developmental stage, snout-vent length varied little across different constant temperatures (F_2, 57_ = 0.72, *P* = 0.49; Figure 4B) whereas, higher constant temperature did lead to increased tail length concordant with constant temperature effects on developmental stage (*F*_2, 57_ = 117.6, *P* < 0.01; Figure 4C). At an average temperature of 20°C moderate DTF had an almost significant, negative effect on snout-vent length (Figure 4B; *F*_1, 37_ = 3.38, *P* = 0.07). Although the mean of the snout-vent lengths of the larvae raised in the DTF treatment was lower, the distribution was wider and encompassed that of the larvae raised at constant 20°C. There was no significant effect of DTF on snout-vent length at an average temperature of 28°C (Figure 4C; *F*_*1*, 40_ = 0.05, *P* = 0.99). However, at an average temperature of 24°C, high levels of DTF did significantly increase average snout-vent length (F_3, 74_ = 9.91, *P* < 0.01; Tukey HSD *P* < 0.01; Figure 4B).

DTF had a significant negative effect on tail length at all three average temperatures (20°C: F_1, 37_ = 66.98, *P* < 0.01; 24°C: F_3, 72_ = 9.60, *P* < 0.01; 28°C: F_1, 40_ = 21.83, *P* < 0.01; Figure 4C). For the 20°C and 28°C treatments, the medium DTF environment had significantly shorter tails than their matched constant temperature treatment groups. However, at average 24°C only, the high DTF environment was observed to have a significant negative effect on tail length (Tukey HSD *P* < 0.01).

### Comparison of predicted and observed effects of DTF

**Figure 5:**
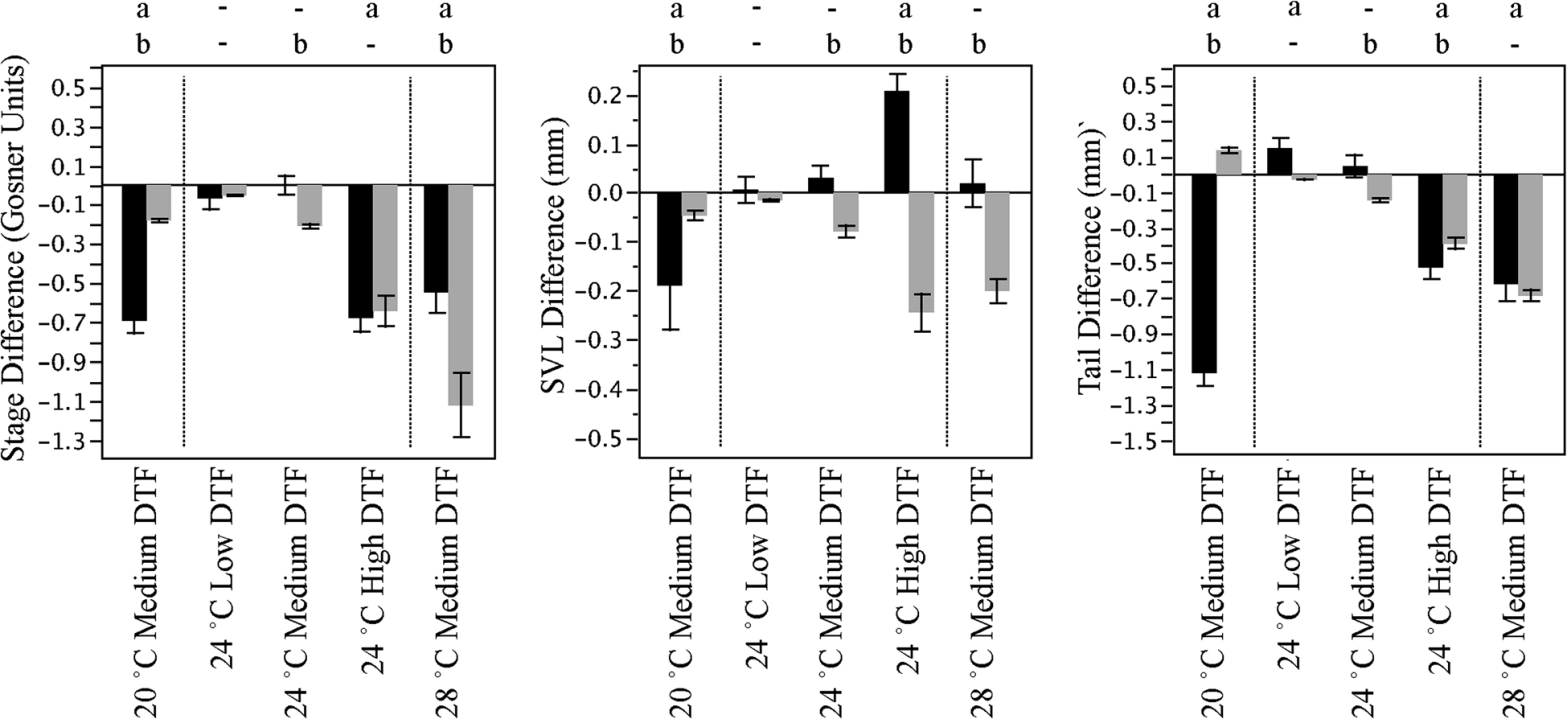
**Comparison of predictions and observations of DTF effects with response at constant temperatures. **Gray bars represent predictions and black bars represent observations. An “**a**” implies a significant deviation of experimental values from their constant temperature counterparts (Tukey HSD P < 0.05) and a “**b**” implies a significant difference between experimental values and theoretically predicted deviations (Tukey HSD P < 0.05).

The effects of variation in DTF predicted from the models that were generated from thermal reaction norms obtained at constant temperatures are all qualitatively similar to those observed in our DTF experiments for developmental stage at 66 hours (Figure 5A). The lack of concordance is quantitative, with larger than predicted declines at an average temperature of 20°C with medium DTF (Tukey HSD *P* < 0.05) and smaller than predicted declines at an average temperature of 28°C with medium DTF. For both the predicted and observed cases the largest effects of DTF occur when embryos experience extreme temperatures (Tukey HSD *P* < 0.05). Low and moderate amounts of DTF at moderate average temperatures have little effect.

In contrast to developmental stage, the predicted effects of variation in DTF on snout-vent length are quite different from those actually observed (Figure 5B). The largest discrepancy is generated by an unexpected increase in snout-vent length at high DTF within the 24°C treatment (Tukey HSD *P* < 0.05), coupled with the lack of sensitivity in the experimental comparisons in the medium and low DTF condition for that same average temperature. In addition, at a low average temperature of 20°C there was a greater than predicted decline in snout-vent length as well (Figure 5B).

The discrepancies between observed and predicted effects of DTF on tail length are quite different from snout-vent length but similar to those seen in developmental rate (Figure 5). In particular, tail length showed a greater decline under medium DTF conditions at 20°C especially in light of the modelling approach predicting a positive effect. The remainder of comparisons between observed and predicted all showed a decline in tail length with increasing DTF culminating in a similar decline for both at 28°C with a medium level of DTF (Tukey HSD *P* < 0.05).

## Discussion

For many amphibians, timing and condition at the transition from non-motile embryonic development to a free-swimming larval phase at hatching is critical in determining offspring survival [32,41,47,48]. Embryonic and larval environments have been shown to influence larval traits such as tail length, snout-vent length, body shape, physiological features (e.g., burst speed; [49-51] and predator escape behaviour [2,38,39,52]. Thus, it is not surprising that the phenotype of a tadpole at the onset of hatching has been shown to affect its probability of surviving predation in the field [2]. In addition, life-history theory predicts that numerous features of the hatchling tadpole phenotype should be under strong selective control [53] and will probably include carryover effects to later stages of development [2,5,54].

Based on our empirical observations [2] and those of others [3,4] and on recent modelling approaches that stem from computation of developmental outcomes as predicted from thermal reaction norms in a variety of ectotherms [6,10,11,55-57], we predicted that DTF would affect developmental and growth rates in our study. Here we show that temperature treatments whose range extended to the maximum or minimum temperatures of this experiment diminished differentiation and tail growth rates. We also found that models derived from data measured in constant temperature environments did not predict the outcomes of DTF for snout-vent length at high temperatures or differentiation rate and tail length at cold temperatures. The high DTF treatment at average 24°C exposed developing larvae to temperatures near their limit of tolerance. Even though the larvae only spent a short time in this region the carryover effects may be substantial. However, the larvae from the moderate DTF treatment at an average of 28°C, which were also exposed to these extreme temperatures, did not show an increase in snout-vent length. This suggests that amount of DTF, not just temperature experienced, also has an effect on development and growth.

In 2006, Kaplan and Phillips [2] showed in a large field study with *B. orientalis* in Korea that a broad range of naturally occurring daily thermal fluctuations both directly impact the probability of surviving predation and interact with maternal effects (i.e., ovum size; see also [26]) in non-additive ways. In that same year Niehaus, Wilson and Franklin [3] compared measures of growth and performance in the laboratory between one level of DTF and one corresponding constant temperature in the Australian marsh frog, *Limnodynastes peronii* and found increased growth throughout development, reduced development time to metamorphosis, and increased post-metamorphic jumping ability, for those embryos and larvae reared in the thermally fluctuating environment. Thus, there is evidence that naturally occurring DTF is important in many aspects of amphibian development. Later studies [5] found that early exposure to DTF did not tend to change sensitivity (i.e., carryover) of physiological function later in development but punctuated the important need to come to understand the role of fluctuating thermal environments “under naturalistic thermal conditions” when considering thermal adaptation and evolution [6]. In addition, Meřaková & Gvoždík [4] also recently explored three levels of DTF at one average temperature in the European newt, *Triturus alpestris*. They found that higher DTF during embryogenesis increased swimming speed at the hatching stage relative to development at lower DTF, but only during a performance test at a cooler temperature. Thus, prior results (and those reported here) show important effects of development in a DTF environment that are dependent on the particular temperatures experienced, the range of temperatures experienced, the developmental stages at which they are experienced, and species.

Studies in insects and reptiles that have relied on empirical modelling approaches have also found that the effects of DTF depend on the magnitude of this variation combined with variation in average temperature (insects: [12,18]; reptiles: [13,15,16,21,58-63]. In many cases the relationship between temperature and developmental rate (or growth rate) becomes non-linear near the limits of a species thermal tolerance range, and models predict that temperature fluctuations should become ecologically important and less predictable when individuals experience temporary exposure to temperatures near these limits for the species (e.g. [18,64]).

In the case of our study, three conditions, our 20°C medium DTF, 24°C high DTF and 28°C medium DTF treatments (Figure 1) transiently expose developing embryos to extreme temperatures where physiological functions that regulate cellular and organismal homeostasis may become impeded. These extreme temperature fluctuations can uncouple differentiation rates and growth rates and further uncouple growth rates for different body regions resulting in body allometry effects. This offers an important explanation for our prior results of increased egg size (a maternal effect and constrained to impacting snout-vent length primarily) decreasing offspring performance via sprint speed under more extreme thermal conditions so that tail to snout-vent length ratios go down dramatically under extreme transient heat exposure in embryos [2,38,39].

## Conclusions

In this study, the biggest difference between predictions based on data from constant temperature environments and observations from development in fluctuating environments was largest when physiological thermal limits were approached. Overall, we find that the difference in levels of temperature variation is important both because of variability *per se* and because of the physiological temperature limits that alter the trajectory of early development in this frog in ways not predicted by our null model.

Recent anthropogenically caused environmental change, including global changes in temperature regimes, offer numerous opportunities for basic research and challenges for conservation. For example, across its range on the Korean peninsula, *B. orientalis* utilizes a variety of oviposition sites that represent a heterogeneous thermal landscape with respect to both average temperature and DTF. In addition to the yet unclear relationships between thermal fluctuations and global warming, recent diminishment and changes in rice agricultural practices have the potential to dramatically shift the thermal landscape with respect to DTF ([65,66] respectively). Prior field results [2] together with the laboratory studies reported here, show that varying levels of DTF in relation to average temperatures can dramatically affect fitness related traits for *B. orientalis.*

Changing thermal landscapes and their impacts on organism adaptability and concomitant distribution, abundance, and evolution also offer unique opportunities to study the dynamics of local adaptation [67] and the rapidity and intensity of natural selection in promoting adaptation [68,69]. Because ectotherms are particularly vulnerable to the developmental ramifications of temperature changes, studies of amphibian embryos and larvae and their development in fluctuating thermal environments can provide important insights towards these goals (see [70]). Amphibians can play an important role in advancing both the theoretical understanding of the development and evolution of thermal adaptation but perhaps more critically in advancing our understanding of amphibian conservation in changing thermal landscapes [6,52,71].

## Abbreviations

DTF: Diel temperature fluctuation.

## Competing interests

The authors declare that they have no competing interests.

## Authors’ contributions

JMA, ESL, and AJ carried out the experimental work. PCP carried out the modelling work. JMA, ESL, AJ, PCP, and RHK analyzed the data. DP provided logistical support with *B. orientalis*. All authors wrote, read and approved the final manuscript.
